# Astrocytic NMDA Receptors in the Basolateral Amygdala Contribute to Facilitation of Fear Extinction

**DOI:** 10.1093/ijnp/pyab055

**Published:** 2021-08-07

**Authors:** Gajanan P Shelkar, Jinxu Liu, Shashank M Dravid

**Affiliations:** Department of Pharmacology and Neuroscience, Creighton University School of Medicine, Omaha, Nebraska, USA

**Keywords:** Astrocytic NMDA receptors, basolateral amygdala, fear extinction, GluN2C-containing NMDA receptors

## Abstract

**Background:**

Enhancement of N-methyl-D-aspartate (NMDA) receptor function using glycine-site agonist D-cycloserine is known to facilitate fear extinction, providing a means to augment cognitive behavioral therapy in anxiety disorders. A novel class of glycine-site agonists has recently been identified, and we have found that the prototype, AICP, is more effective than D-cycloserine in modulating neuronal function.

**Methods:**

Using novel glycine-site agonist AICP, local infusion studies, and genetic models, we elucidated the role of GluN2C-containing receptors in fear extinction.

**Results:**

We tested the effect of intracerebroventricular injection of AICP on fear extinction and found a robust facilitation of fear extinction. This effect was dependent on GluN2C subunit, consistent with superagonist action of AICP at GluN2C-containing receptors. Local infusion studies in wild-type and GluN2C knockout mice suggested that AICP produces its effect via GluN2C-containing receptors in the basolateral amygdala (BLA). Furthermore, consistent with astrocytic expression of GluN2C subunit in the amygdala, we found that AICP did not facilitate fear extinction in mice with conditional deletion of obligatory GluN1 subunit from astrocytes. Importantly, chemogenetic activation of astrocytes in the basolateral amygdala facilitated fear extinction. Acutely, AICP was found to facilitate excitatory neurotransmission in the BLA via presynaptic GluN2C-dependent mechanism. Immunohistochemical studies suggest that AICP-mediated facilitation of fear extinction involves synaptic insertion of α-amino-3-hydroxy-5-methyl-4-isoxazole propionic acid (AMPA) receptor GluA1 subunit.

**Conclusion:**

These results identify a unique role of astrocytic NMDA receptors composed of GluN2C subunit in extinction of conditioned fear memory and demonstrate that further development of recently identified superagonists of GluN2C-containing receptors may have utility for anxiety disorders.

**Significance Statement**

Fear extinction is a learned inhibitory response to conditioned fear when the initially noxious conditioned responses are no longer associated with aversive outcomes, and it is an important model of cognitive-behavioral therapy for human anxiety disorders. The basolateral amygdala (BLA) is one of the brain nuclei that play an essential role in fear extinction. We recently identified predominant expression of GluN2C-containing NMDA receptors in astrocytes in BLA; however, their functional roles remain poorly understood. Here, by using local infusion studies and genetic models, we demonstrate that the facilitation of GluN2C-containing NMDA receptors in the BLA by novel glycine site agonist AICP (highly effective than US-FDA–approved D-cycloserine), facilitates fear extinction. Furthermore, AICP treatment enhances excitatory neurotransmission and strengthens excitatory synapses in the BLA. Overall, the present study identifies a novel target, “GluN2C-containing NMDA receptors in astrocytes,” for facilitating fear extinction, which may have important translational implications for PTSD and anxiety-like disorders.

## Introduction

Anxiety disorders are the most common form of psychiatric disorders, with an incidence of 18% and a lifetime prevalence of 30% (Kessler et al., 2005). The most common forms of anxiety disorders include panic disorders, obsessive compulsive disorder, social anxiety disorders, specific phobias, and post-traumatic stress disorder (PTSD). Therapeutic interventions for these disorders primarily include selective serotonin reuptake inhibitors and some form of cognitive behavioral therapy (CBT), but their effectiveness is limited. Therapies that can effectively augment CBT may provide a new avenue for treating anxiety disorders. One of the behavioral paradigms used to test some aspects of anxiety disorders is fear conditioning in which a neutral stimulus, such as an acoustic tone (the conditional stimulus, or CS), is paired with a noxious unconditional stimulus (US), such as a foot shock. After only a few conditioning trials, the CS comes to evoke a learned fear response such as freezing. Conditioned fear can be extinguished if the animal is exposed to a previously trained CS in the absence of the US and is termed fear extinction (Falls et al., 1992; Rescorla, 2001; Pavlov, 2010).

The finding that D-cycloserine (DCS), a glycine site agonist of N-methyl-D-aspartate (NMDA) receptors, can facilitate fear extinction in rodents, a process that shares conceptual similarity to CBT in humans, was instrumental in opening a new avenue for treatment of anxiety disorders. Systemic administration of D-cycloserine (DCS), an NMDA receptor glycine site agonist, before or immediately after extinction training, facilitates extinction of fear in rodents (Cox and Westbrook, 1994; Baker and Azorlosa, 1996; Fanselow and LeDoux, 1999; Royer and Paré, 2002; Goosens and Maren, 2004; Sotres-Bayon et al., 2007, 2009). DCS has also been tested in clinical trials for PTSD but was only found to have modest efficacy (Mataix-Cols et al., 2017; McGuire et al., 2017), suggesting the need for further development of DCS-like molecules to enhance clinical efficacy. DCS is a GluN1 glycine site agonist (Lee et al., 2006; Woods and Bouton, 2006; Weber et al., 2007), but its efficacy compared with the endogenous agonists glycine and D-serine is GluN2-subunit dependent (Chang and Maren, 2011; Gupta et al., 2013; Sierra et al., 2016). DCS is a superagonist at GluN1/2C receptors with an efficacy of approximately 190% compared with endogenous glycine or D-serine (Ren et al., 2013; Sierra et al., 2016). Recently, AICP, a superagonist with approximately 350% efficacy compared with glycine at GluN1/2C receptors has been discovered (Jessen et al., 2017). We have found that AICP produces GluN2C-dependent actions both in electrophysiology recordings as well as in vivo (Liu et al., 2019); however, its efficacy for facilitating fear extinction is unknown.

Here, we tested the efficacy of AICP in fear extinction and addressed its mechanism of action using genetic models. Interestingly, we found that AICP facilitates fear extinction, and its action is dependent on astrocytic GluN2C-containing receptors in the basolateral amygdala (BLA). In support of an astrocytic role in fear extinction, additional data demonstrated that chemogenetic activation of BLA astrocytes also facilitated fear extinction. Together, these studies identify a novel target for facilitation of fear extinction, which has implications for PTSD and other anxiety disorders.

## METHODS AND MATERIALS

### Animals

We used wild-type (WT) and the Grin2C^tm1(EGFP/cre/ERT2)Wtsi^ (GluN2C KO) mouse line, which were obtained from Wellcome Trust Sanger Institute, on pure C57BL/6N background (Ravikrishnan et al., 2018; Shelkar et al., 2019). The Grin1^flox/flox^ (or GluN1^flox/flox^) mice (congenic C57BL/6, 005246 Jackson Labs) were crossed with Aldh1l1cre/ERT2 (congenic C57BL/6, 031008 Jackson Labs, Bar Harbor, ME, USA) driver mice with tamoxifen-inducible cre recombinase expression directed at astrocytes. The mice of both the sexes were used for experiments. Two- to 3-month-old mice were used for stereotaxic cannulations and virus injection. Mice were housed at a constant temperature (22 ± 1°C) and a 12-h-light/-dark cycle with ad libitum access to food and water as previously described (Shelkar et al., 2019). While AAV injected mice were group housed, the mice implanted with guide cannula were housed singly to prevent fighting- or grooming-induced retraction of cannula. All procedures were approved by the Creighton University Institutional Animal Care and Use Committee and conformed to the NIH Guide for the Care and Use of Laboratory Animals.

### Drugs

D-cycloserine (Sigma-Aldrich, St. Louis, MO, USA) was dissolved in 0.9% saline solution, and the stock of AICP (a generous gift from Dr Rasmus Clausen, University of Copenhagen) was prepared in DMSO. This stock was diluted further to the final working concentration with PEG400 (0.5–1% v/v DMSO in PEG400). AICP was administered at the dose of 3.5 µg in 1 µL (ICV) and 100 ng in 0.5 µL (BLA or mediodorsal thalamus [MDT]) over a period of 2–3 minutes. The injection cannula was left in a place for an additional 2 minutes and was slowly withdrawn. Tamoxifen (Sigma-Aldrich) was dissolved in corn oil (Sigma-Aldrich) at a concentration of 20 mg/mL by shaking overnight at 37°C and administered by intraperitoneal (i.p.) route Clozapine N-oxide (CNO) dihydrochloride (Hello Bio Inc., Princeton, NJ, USA) was dissolved in saline to the final concentration and administered i.p. in a 1-mg/kg dose.

### Surgical Procedures

For stereotaxic cannulations and virus injections, mice were anesthetized with isoflurane and placed in a stereotaxic frame, the skull was opened, and a small hole was drilled through the skull at stereotaxic co-ordinates for the lateral ventricle (ICV, AP: −0.22 mm, ML: 0.8 mm, DV: −2.3 mm), MDT (AP: −1.5 mm, ML: ±0.3 mm, DV: −3 mm), or BLA (AP: −1.7, ML: ±3.1, DV: −3.8) (Paxinos and Franklin, 2001). The guide cannula prepared in house was slowly lowered at the appropriate length and was secured to the skull using mounting screws and dental acrylic cement. Mice were implanted with either unilateral (ICV) or bilateral (BLA and MDT) cannula. A stainless-steel dummy cannula was used to occlude the guide cannula when not in use. The animals were then allowed to recover for 10 days before initiation of experiments.

For virus injections, Aldh1l1-cre/ERT2 mice were anaesthetized, and a small hole was drilled above the BLA (−1.7 mm posterior, ±3.15 mm lateral, and −3.8 mm ventral with respect to bregma). The virus particles AAV2/DJ8-CAG-DIO-hM3D (Gq)-mCherry (Canadian Neurophotonics Platform, Québec, Canada) were injected (200 nL) bilaterally into BLA as described previously (Liu et al., 2019). After 2 weeks of surgery, these mice were injected with tamoxifen (75 mg/kg, ip) once per day for 5 days; 4 weeks after last tamoxifen injection, they were used for behavioral assay. The 4-week duration was allowed for tamoxifen inducible cre recombination and cre-dependent virus expression. After the end of behavioral experiments, the brains from all the animals were verified for cannula location and viral expression by examining the fixed brain tissue from these animals under light or fluorescent microscope. The animals showing cannula placement outside the BLA in surrounding regions were excluded from the study.

### Fear Extinction

Fear extinction was conducted as previously described with some modifications (Hillman et al., 2011). The freeze monitor system consisted of a Plexiglas rodent conditioning chamber (model 2325-0241 San Diego Instruments, San Diego, CA, USA) with a metal grid floor for foot shock. A light/sound stimulus unit was enclosed in a sound-attenuating chamber. White noise was provided in each isolation cabinet with a fan. A video-camera (Logitech) was mounted at the top of each isolation chamber to videotape all sessions. After surgical recovery, mice were habituated to the experimental room and fear conditioning chamber. On day 1 (fear conditioning), mice were conditioned (context-A) with 5 CS-US trials. The chamber was cleaned with a 70% ethanol solution, scented with linen-scented air freshener, and illuminated with a 40-watt white light. The fan mounted on the cabinet provided white noise. Each trial consisted of 30 seconds of tone (CS, 85 dB) that co-terminated with 2 seconds of a 0.5-mA foot shock (US) with an inter-trial interval of 1 minute. On day 2, fear extinction training was carried out in a novel context. The context of the chamber was altered by including different cues on the wall, changing the floor texture, and including a different scent (1% vanilla solution in 70% ethanol, context-B). Extinction training consisted of 4 tone-alone (85 dB) trials each of 2 minutes with an inter-trial interval of 1 minute. Only 4 extinction US-only trials were given to limit training-induced reduction in fear response, allowing better evaluation of AICP effect. Vehicle or AICP (3.5 µg in 1 µL, ICV), AICP (100 ng in 0.5 µL, intra-BLA or intra-MDT) or DCS (62 µg in 1 µL, ICV), or CNO (1 mg/kg, i.p.) was administered immediately after extinction training. To delineate whether the effect of AICP depends on extinction training, we performed control experiments wherein animals received AICP (3.5 µg in 1 µL, ICV) treatment without exposing them to extinction training. On day 3, the post-extinction test was carried out in the same context as extinction training (context-B). Mice were tested for freezing response to a single 2-minute tone (85 dB). We opted for the ABB design to test the effect of AICP on extinction of cue-specific freeing behavior, and therefore the recall test was conducted in the same context wherein extinction training was performed. All the video-taped behavioral sessions were scored manually by an observer blinded to treatments.

### Electrophysiology

Whole-cell electrophysiology was performed as previously described (Gupta et al., 2016; Liu et al., 2019, 2020). Mice were decapitated under isoflurane anesthesia, and brains were removed rapidly and placed in ice-cold artificial cerebrospinal fluid of the following composition (in mM): 130 NaCl, 24 NaHCO_3_, 3.5 KCl, 1.25 NaH_2_PO_4_, 2.4 CaCl_2_, 2.5 MgCl_2_, and 10 glucose saturated with 95% O_2_/5% CO_2_. A dose of 300-μm-thick coronal sections was prepared using vibrating microtome (Leica VT1200, Buffalo Grove, IL, USA). Whole-cell patch recordings were obtained from BLA or MDT neurons in voltage-clamp or current-clamp configurations with an Axopatch 200B (Molecular Devices, Sunnyvale, CA, USA). Glass pipettes with a resistance of 3–5 mOhm were used. Signal was filtered at 2 kHz and digitized at 10 kHz using an Axon Digidata 1440A analog-to-digital board (Molecular Devices). Whole-cell recordings with a pipette access resistance <20 mOhm and that changed <20% during the duration of recording were included. For voltage-clamp recordings, glass pipettes were filled with an internal solution consisting of (in mM) 126 cesium methanesulfonate, 8 NaCl, 10 HEPES, 8 Na_2_-phosphocreatine, 0.3 Na_2_GTP, 4 MgATP, 0.1 CaCl_2_, and 1 EGTA (pH 7.3). Then 2.9 mM QX-314 was added to block voltage-gated sodium channels in recorded cells. Miniature excitatory postsynaptic currents (mEPSCs) were recorded at −70 mV in the presence of 0.5 µM tetrodotoxin and 100 µM picrotoxin. The mEPSC recordings were analyzed using Minianalysis software (Synaposoft, Atlanta, GA, USA) with an amplitude threshold set at 5 pA. The frequency of the miniature currents was determined. The maximal response from each cell following AICP application was selected, averaged, and used for analyses. For current-clamp recordings, patch pipettes were filled with a solution containing (in mM) 105 K-gluconate, 30 KCl, 10 HEPES, 10 Na_2_-phosphocreatine,4 Na_2_ATP, and 0.3 Na_2_GTP (pH 7.2). The current steps were applied for 1 second in 20-pA increments from −100 pA to 100 pA. Spike frequency was calculated in hertz as a ratio between number of spikes and current step duration.

### Immunohistochemistry

Immunohistochemistry was performed in vehicle- or AICP-treated mice as previously described (Ravikrishnan et al., 2018; Liu et al., 2019). Briefly, immediately following post-extinction test, vehicle- or AICP-treated mice were transcardially perfused and brains were removed and cryoprotected. The brains were cut in a coronal plane and sections passing through BLA were collected. Sections were washed with PBT (0.25% Triton-X in 0.01 M PB) and incubated in appropriate blocking solution containing 10% normal goat serum or normal donkey serum in PBT for 1 hour at room temperature. Following blocking, sections were incubated in primary antibodies at appropriate concentrations (rabbit anti-GluA1, 1:250, Millipore 05-855R; mouse anti-PSD95, 1:300, Invitrogen MA1-046; goat anti-bassoon, 1:150, Novus Biologicals NBP1-46351) in solution containing 5% normal goat serum and/or donkey serum in PBT overnight at 4°C. On the next day, sections were washed and thereafter incubated with the appropriate secondary antibodies conjugated to AlexaFluor 488 (donkey anti-mouse 1:500 in PBT), AlexaFluor 594 (donkey anti-rabbit 1:500 in PBT), AlexaFluor 647 (goat anti-rabbit 1:500 in PBT), or AlexaFluor 647 (donkey anti-goat 1:500 in PBT) for 2 hours at room temperature. Sections were then washed and mounted with Fluoromount-G (SouthernBiotech, Birmingham, AL, USA). Images were acquired with a Nikon Ti-E inverted microscope with a Yokagawa spinning disc for confocal imaging scanning confocal microscope. For puncta analysis, images of equivalent regions, 1024 × 1024 pixels, were captured using a 60×, oil-immersion objective at a 1× zoom. The BLA was scanned at 0.3-μm intervals along the z-axis and an optical section (7.2 μm thick) was taken from each tissue section. Co-localization and the puncta number were analyzed by Volocity (PerkinElmer Inc., Coventry, UK). A total of 22–24 images were analyzed from each group of AICP-treated (n = 5 mice) and vehicle-treated (n = 3 mice) animals. The data were collated and used for statistical analysis.

## RESULTS

### Intracerebroventricular Injection of GluN1/2C Superagonist AICP Facilitates Fear Extinction

**Figure 1.**
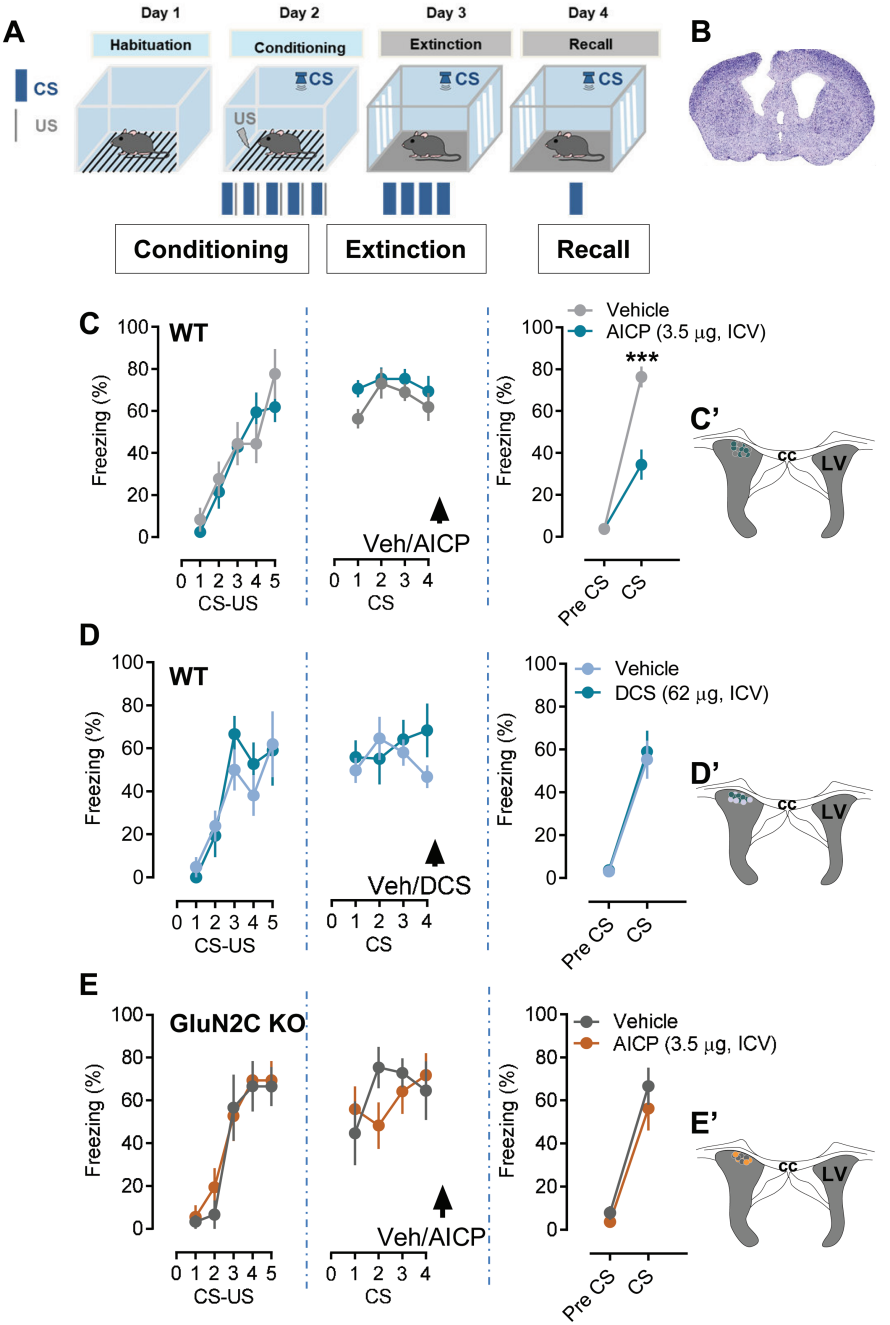
Intracerebroventricular (ICV) injection of AICP facilitates fear extinction. (A) Schematic of experimental design used for fear conditioning and fear extinction. CS, conditioned stimulus; US, unconditioned stimulus. (B) Schematic of injection site and verification of guide cannula targeted at lateral ventricle (LV). (C) Effect of AICP on fear extinction in WT mice. ICV injection of AICP (3.5 µg) immediately after extinction training significantly reduced freezing behavior in post-extinction recall test (vehicle n = 6, AICP n = 7). Two-way ANOVA showed significant effect of treatment (F [1, 11] = 91.16, *P* < .0001) and interaction (F [1, 11] = 15.14, *P* = .0025). Post-hoc Bonferroni’s test showed significance effect of AICP on freezing behavior during post-extinction recall test (vehicle 76.387 ± 5.009 vs AICP 34.402 ± 7.227, *P* < .0001). (D) Effect of DCS (62 µg, ICV) on fear extinction in WT mice. Administration of DCS immediately after extinction training did not show any significant effect in recall test (vehicle 55.355 ± 8.896 vs DCS 59.025 ± 9.826; *P* > .9999; n = 7 vehicle, 6 DCS; two-way ANOVA with Bonferroni’s post hoc test). (E) AICP (3.5 µg, ICV) treatment in GluN2C KO mice did not show any significant effect on freezing behavior in recall test (vehicle 66.666 ± 8.640 vs AICP 56.248 ± 10.134; *P* = .6261; n = 5 vehicle, 6 AICP; two-way ANOVA with Bonferroni’s post hoc test). The insets C’, D’, and E’ from respective C, D, and E figures show the injection placement site in the LV from the respective vehicle and AICP or vehicle and DCS treatments. Each circle represents an individual animal. Data from the animals showing injection cites within LV were used for analysis. Data were represented as mean ± SEM.

We tested the effect of AICP on fear extinction. To eliminate potential pharmacokinetic and blood-brain barrier permeability issues, we assessed the effect of ICV injection of AICP. We used a shorter extinction training protocol to better evaluate drug-induced facilitation of fear extinction (Figure 1A). ICV injection of AICP was conducted immediately after fear extinction training because DCS administration in clinical trials has been found to be effective after a successful CBT. AICP dose was based on our previous evaluation of AICP effect in vivo (Liu et al., 2019). Administration of the AICP (3.5 µg in 1 µL) significantly reduced the level of freezing during post-extinction recall test, demonstrating that AICP facilitates fear extinction (Figure 1C; n = 6 vehicle, 7 AICP, *P* = .0001, 2-way ANOVA with Bonferroni’s test). Under the same experimental conditions, DCS (62 µg in 1 µL) was ineffective in reducing freezing, suggesting a stronger effect of AICP on fear extinction compared with DCS (Figure 1D; n = 7 vehicle, 6 DCS, *P* = .8568, 2-way ANOVA with Bonferroni’s test). No significant differences in baseline freezing before CS presentation during post-extinction recall test were observed in vehicle-, AICP-, or DCS-treated animals (*P* > .05 each). Further, AICP (3.5 µg, ICV) did not show any significant effect in the group of animals that do not receive extinction training, suggesting that AICP does not have its per-se effect and requires interactive behavioral extinction training to produce its effects (*P* > .05; supplementary Figure 1A). We next addressed whether AICP (3.5 µg) effects on fear extinction are dependent on the GluN2C subunit. Fear extinction experiments were performed in GluN2C KO mice. AICP was ineffective in facilitating fear extinction in GluN2C KO, with no difference in the vehicle and AICP groups in the post-extinction recall test, demonstrating the requirement for GluN2C in AICP effect on fear extinction (Figure 1E; n = 5 vehicle, 6 AICP, *P* = .6787, 2-way ANOVA with Bonferroni’s test). No changes in baseline freezing before CS presentation during post-extinction recall test were observed following vehicle or AICP treatment in GluN2C KO mice

### Infusion of AICP Into the Basolateral Amygdala but Not Mediodorsal Thalamus Facilitates Fear Extinction

**Figure 2.**
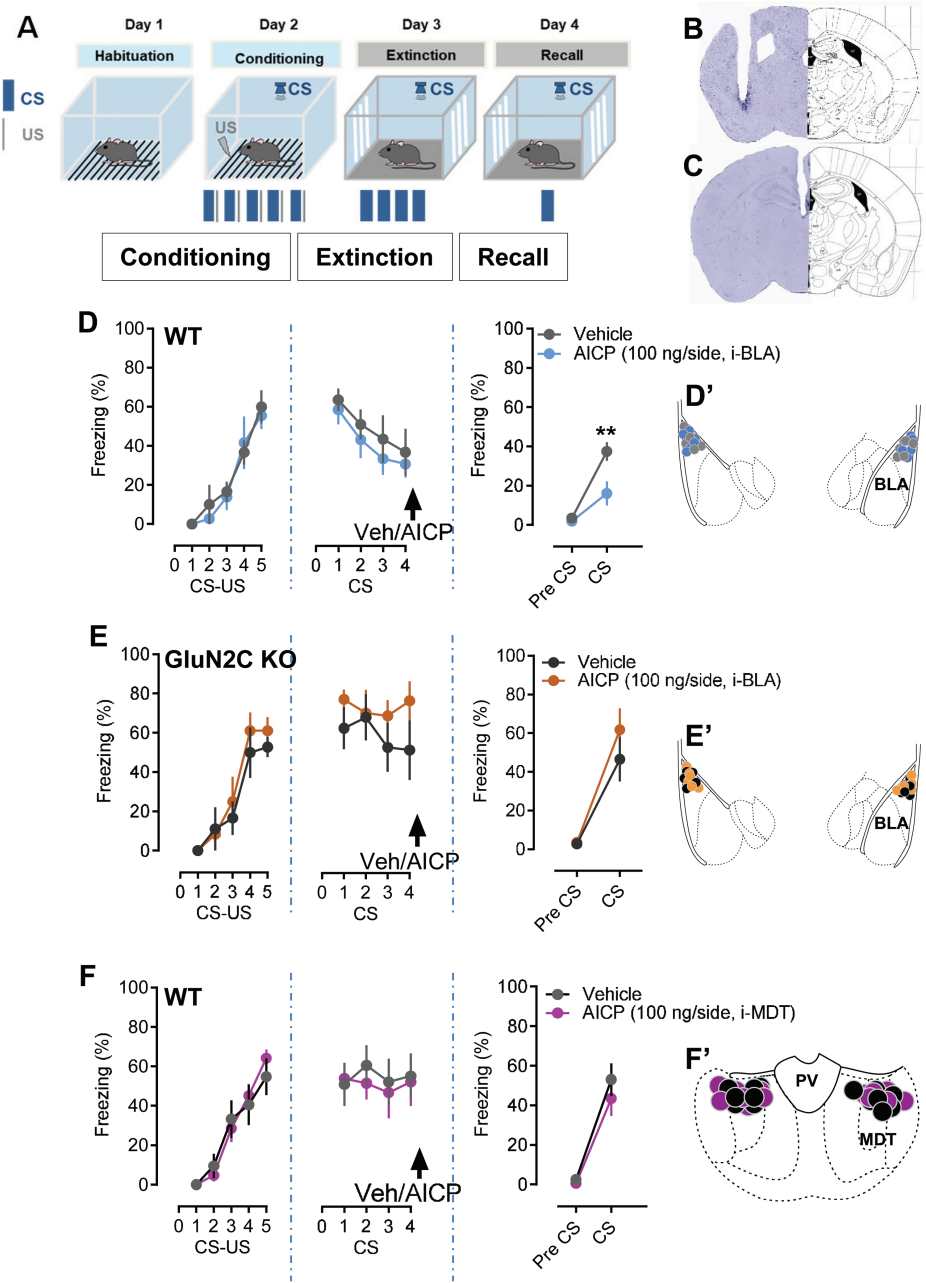
Facilitation of GluN2C-containing N-methyl-D-aspartate (NMDA) receptors in basolateral amygdala (BLA), but not mediodorsal thalamus (MDT), reduces freezing behavior in recall test. (A) Experimental design used for fear conditioning and fear extinction. CS, conditioned stimulus; US, unconditioned stimulus. (B) Verification of guide cannula targeted at BLA. (C) Verification of the guide cannula into MDT. (D) Intra-BLA injection of AICP (100 ng/side) immediately after extinction training significantly reduced freezing behavior in post-extinction recall test in WT animals. Two-way ANOVA showed significant effect of treatment (F [1, 10] = 6.495, *P* < .0289) and interaction (F [1, 10] = 5.964, *P* = .0347). Post-hoc Bonferroni’s test showed significance effect of AICP on freezing behavior during post-extinction recall test (vehicle 37.496 ± 4.751 vs AICP 16.070 ± 6.128, *P* = .0042; n = 5 vehicle, 6 AICP). AICP (100 ng/side) treatment in GluN2C KO mice did not show any significant effect (E) (vehicle 46.522 ± 11.457 vs AICP 61.802 ± 11.151; *P* = .3937; n = 6 vehicle, 6 AICP; two-way ANOVA with Bonferroni’s post hoc test). (F) Intra-MDT administration of AICP (100 ng/side) immediately after extinction training did not show any significant effect in recall test (vehicle 53.020 ± 8.224 vs AICP 43.450 ± 8.856; *P* = .5599; n = 7 vehicle, 7 AICP; two-way ANOVA with Bonferroni’s post hoc test). The insets D’ and E’ from respective D and E figures show the injection placement site in the BLA from the respective vehicle and AICP treatments. The insets F’ show the injection placement site in the MDT from vehicle and AICP treatments. Each circle represents an individual animal. Data from the animals showing injection cites within BLA or MDT were used for analysis. Data were represented as mean ± SEM.

The neurocircuitry in the BLA has been demonstrated to be critical for fear learning (Duvarci and Pare, 2014; Janak and Tye, 2015). In addition, the GluN2C subunit is enriched in the MDT, which has also been found to contribute to fear extinction (Lee et al., 2011; Ravikrishnan et al., 2018). Lee and colleagues have found that the tonic firing of MDT is critical in modulation of fear extinction (Lee et al., 2011). Additionally, thalamic reticular nucleus neurons suppress the spiking activity of the medial part of the dorsal midline thalamus, and a blockade of this inhibitory pathway disrupted fear extinction (Lee et al., 2019). Therefore, we also examined the role of GluN2C subunits in MDT in fear extinction. Using the local infusion technique, we explored the site of action of AICP on fear extinction. We found that AICP infusion into the BLA immediately after extinction training reduced freezing in the recall test, indicating facilitation of fear extinction (Figure 2A–C; n = 5 vehicle, 6 AICP, *P* = .0125, 2-way ANOVA with Bonferroni’s test). However, infusion of AICP into the MDT did not produce an effect on freezing in the post-extinction test (Figure 2A and F; n = 7 vehicle, 7 AICP, *P* = .5599, 2-way ANOVA with Bonferroni’s test). We also tested whether the effect of AICP in the BLA was dependent on GluN2C-containing receptors by conducting experiments in GluN2C KO mice. Similar to our findings in the ICV studies local infusion of AICP into the BLA of GluN2C KO mice did not facilitate fear extinction, demonstrating that the AICP effect in the BLA is mediated by GluN2C-containing receptors (Figure 2D; n = 6 vehicle, 6 AICP, *P* = .6087, 2-way ANOVA with Bonferroni’s test).

### AICP Effect on Fear Extinction Is Dependent on NMDA Receptors in Astrocytes

**Figure 3.**
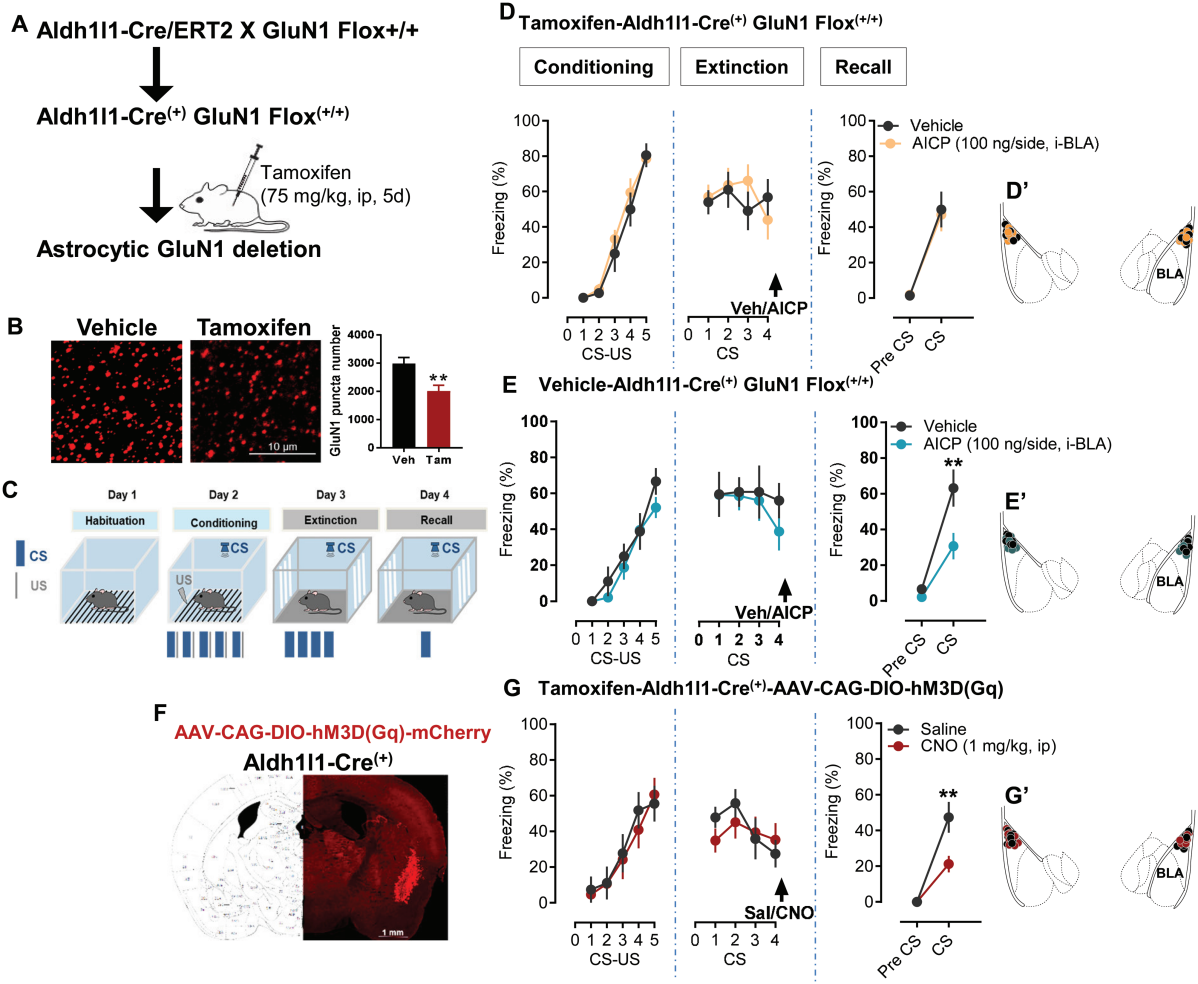
Facilitation of fear extinction by AICP is dependent on astrocytic N-methyl-D-aspartate (NMDA) receptors. (A) Breeding strategy used for conditional deletion of astrocytic NMDA receptors by making use of tamoxifen-inducible cre recombinase in astrocytes. (B) Immunohistochemical analysis showing reduction of GluN1 puncta in basolateral amygdala (BLA) of tamoxifen-treated mice demonstrating astrocytic deletion of GluN1. (C) Experimental approach adopted for fear conditioning and extinction experiments. CS, conditioned stimulus; US, unconditioned stimulus. (D) Effect of AICP in tamoxifen-treated Aldh^Cre/ERT2^GluN1^Flox(+/+)^ mice. Intra-BLA injection of AICP (100 ng/side) immediately after extinction training in astrocytic GluN1-deleted mice (tamoxifen injected group) fails to produce significant effect in post-extinction recall test (vehicle 49.995 ± 10.093 vs AICP 47.020 ± 9.348; *P* > .9999; n = 6 vehicle, 7 AICP; two-way ANOVA with Bonferroni’s post hoc test). (E) Effect of AICP in vehicle (corn oil)-treated Aldh^Cre/ERT2^GluN1^Flox(+/+)^ mice. Intra-BLA injection of AICP (100 ng/side) immediately after extinction training in astrocytic GluN1 intact mice (vehicle-injected group) showed robust facilitation of fear extinction. Two-way ANOVA showed significant effect of treatment (F [1, 12] = 7.398, *P* = .0186) and interaction (F [1, 12] = 5.588, *P* = .0358). Post-hoc Bonferroni’s test showed a significant effect of AICP on freezing behavior during post-extinction recall test (vehicle 63.192 ± 10.398 vs AICP 30.725 ± 7.426, *P* = .0029; n = 6 vehicle, 8 AICP). (F) Schematic showing site of virus injections. Coronal image from ALDH1l1^cre/ERT2^ mouse injected with AAV-CAG-DIO-hM3D(Gq)-mCherry showing virus expression in BLA. (G) Immediately following fear extinction training, DREADD-injected mice were administered with saline or CNO (1 mg/kg, ip). A significant reduction in freezing behavior in post-extinction recall test was observed in CNO-treated mice compared with the saline treatment two-way ANOVA, which showed significant effect of treatment (F [1, 15] = 7.582, *P* = .0148) and interaction (F [1, 15] = 7.582, *P* = .0148). Post-hoc Bonferroni’s test showed a significant effect of CNO on freezing behavior during post-extinction recall test (saline 47.393 ± 8.624 vs CNO 21.292 ± 4.630, *P* = .0010; n = 8 vehicle, 9 CNO). The insets D’ and E’ from the respective D and E figures show the injection placement site in BLA from the respective vehicle and AICP treatments in tamoxifen- or vehicle-treated Aldh^Cre/ERT2^GluN1^Flox(+/+)^ mice. G’ shows the virus injection cites in BLA from tamoxifen-treated ALDH1l1^cre/ERT2^ (saline and CNO) mice. Each circle represents an individual animal. Data from the animals showing injection cites within BLA were used for analysis. Data were represented as mean ± SEM.

We have recently shown that GluN2C subunit is enriched in astrocytes in the majority of corticolimbic regions, including BLA (Ravikrishnan et al., 2018), which is the site for synaptic plasticity relevant to associative fear learning. Thus, it is possible that GluN2C-containing NMDA receptors in astrocytes are responsible for the AICP effect. To test this hypothesis, we used a conditional knockout (KO) strategy. We used Aldh1L1-CreERT2 mice, which allow expression of tamoxifen-inducible cre recombinase exclusively in astrocytes (Srinivasan et al., 2016). Aldh1L1-CreERT2 mice were crossed with GluN1^flox/flox^ mice to generate mice with conditional deletion of the obligatory GluN1 subunit. Immunohistochemistry analysis of BLA in tamoxifen- vs vehicle-injected animals found a significant reduction in GluN1 puncta in mice injected with tamoxifen, demonstrating astrocytic deletion of GluN1 (Figure 3A–B). Effect of AICP injection in the BLA on fear extinction was evaluated in Aldh11l-^CreERT2^GluN1^flox/flox^ mice injected with tamoxifen or vehicle. In tamoxifen-injected mice, which leads to deletion of the astrocytic GluN1, AICP was ineffective in facilitating fear extinction (Figure 3D; n = 6 vehicle, 7 AICP, *P* > .9999, 2-way ANOVA, Bonferroni’s test). In contrast, AICP produced robust facilitation of fear extinction in the i.p. vehicle group with no deletion of astrocytic GluN1 (Figure 3E; n = 5 vehicle, 7 AICP, *P* = .0029, 2-way ANOVA with Bonferroni’s test). We also compared whether there are differences in fear conditioning and extinction in mice on ablation of NMDA receptors from astrocytes. No significant differences were observed in fear conditioning, extinction (*P* = .4614, *P* = .5887 respectively, 2-way ANOVA), or recall (*P* = .3839, unpaired *t* test) between the i.p. tamoxifen/vehicle control groups, suggesting that deletion of astrocytic NMDA receptor alone does not impair fear learning. No change in baseline pre-CS freezing before CS presentation during the post-extinction recall test was observed in any of the treatment groups. Together, this set of experiments demonstrated that the AICP effect on fear extinction is dependent on astrocytic NMDA receptors in the BLA.

### Chemogenetic Activation of BLA Astrocytes Facilitates Fear Extinction

To further establish a role of BLA astrocytes in fear extinction, we used a chemogenetic approach. Aldh1l1-CreERT2 mice were injected with AAV-CAG-DIO-hM3D(G_q_)-mCherry virus. After a sufficient interval, cre expression was induced by tamoxifen injection. After allowing time for cre-dependent DREADD expression, the effect of i.p. administration of CNO or saline immediately after extinction training was evaluated. The CNO-treated (1 mg/kg, i.p.) group showed significantly lower freezing compared with the saline group in post-extinction recall testing (Figure 3G; n = 8 saline, 10 CNO, *P* = .0010, 2-way ANOVA with Bonferroni’s test), demonstrating that activation of BLA astrocytes facilitates fear extinction. CNO (1 mg/kg, i.p.) failed to produce any significant changes in freezing behavior in recall test in the absence of DREADD expression demonstrating lack of non-specific effects (Supplementary Figure 1B). DREADD expression was confirmed by examining the mCherry reporter in the BLA (Figure 3F).

### AICP Increases Excitatory Neurotransmission in BLA Neurons

**Figure 4.**
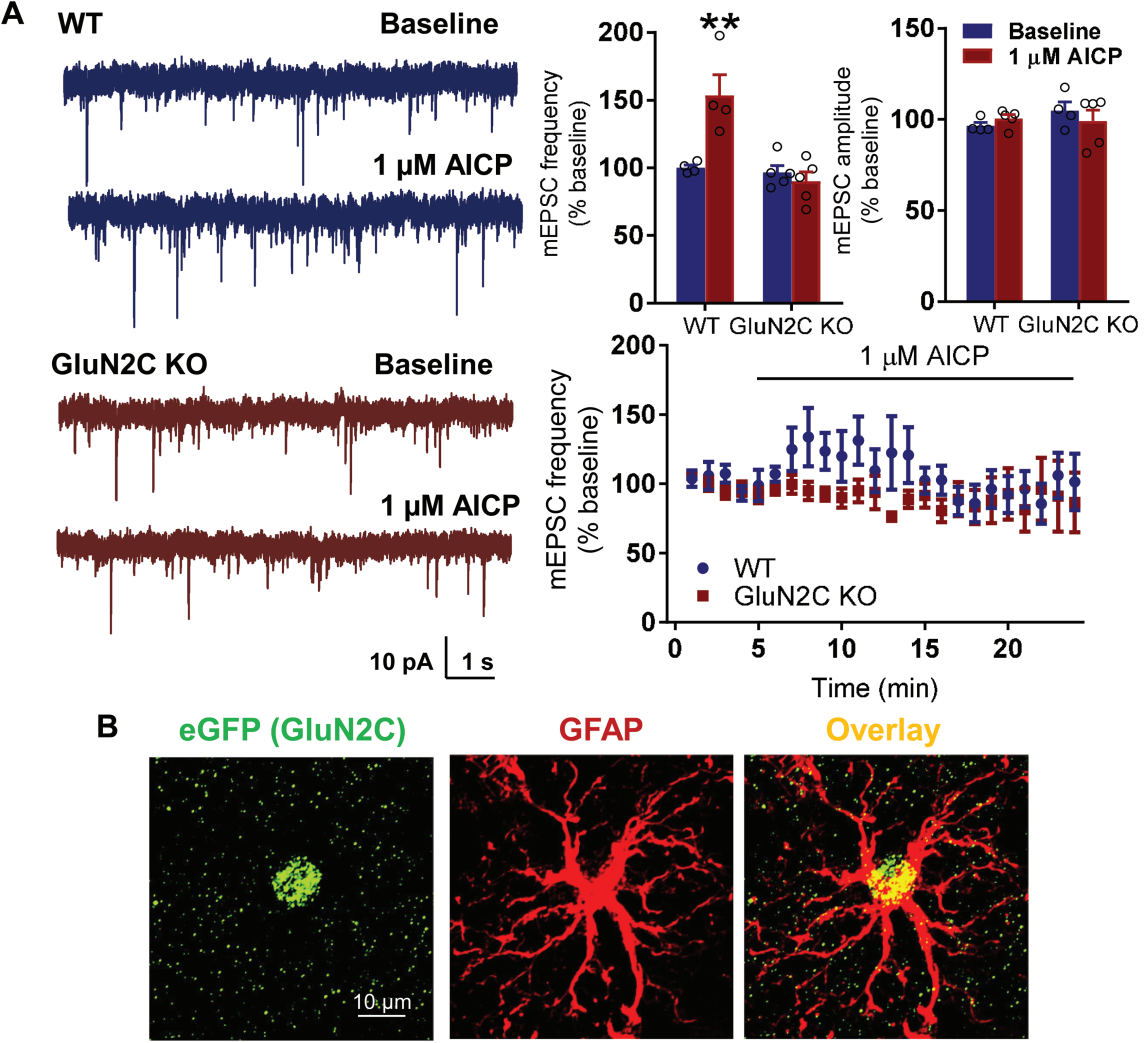
AICP increase excitatory neurotransmission in basolateral amygdala (BLA) neurons. (A) Whole cell voltage clamp recordings from BLA neurons were conducted in WT and GluN2C KO mice. A significant increase in the frequency of mEPSC was observed following bath application of AICP in WT mice (WT-baseline 100.117 ± 2.021 vs WT-1µM AICP 153.415 ± 15.345, *P* = .0016; two-way ANOVA with Bonferroni’s post hoc test). No significant differences were observed in amplitude of mEPSCs following AICP treatment (*P* > .05). AICP failed to produce any significant effect on mEPSC frequency and amplitude in GluN2C KO mice (*P* > .05). Data are represented as mean ± SEM. (B) Immunohistochemical analysis in Grin2C^EGFP-CreERT2^ (GluN2C KO) mouse model. The coronal sections passing through BLA were immunolabelled for eGFP (GluN2C) and GFAP. Expression of eGFP in the Grin2C-reporter model was found to primarily co-localize with astrocytic marker GFAP.

To assess whether AICP modulates excitatory neurotransmission in the BLA, we examined the effect of bath application of AICP on mEPSC. We found a significant increase in mEPSC frequency in WT mice following application of AICP (Figure 4A; WT-baseline 100.1 ± 2.0 vs WT-1 µM AICP 153.4 ± 15.3, *P* = .0016; 2-way ANOVA with Bonferroni’s test). No significant change in amplitude of mEPSC was found with AICP, suggesting a presynaptic mechanism of action of AICP. Interestingly, AICP failed to produce any significant effect in GluN2C KO mice (baseline 96.5 ± 5.1 vs 1 µM AICP 89.9 ± 7, *P* > .9999; 2-way ANOVA with Bonferroni’s test), demonstrating a GluN2C-dependent effect of AICP. We previously reported selective expression of GluN2C subunit in astrocytes in the BLA (Ravikrishnan et al., 2018). We further confirmed this using the reporter model and found that EGFP (GluN2C) selectively colocalized with GFAP (Figure 4B). Because we did not observe any effect of AICP on fear extinction when locally injected into MDT, we examined whether lack of behavioral effect translated to lack of effect on neuronal function. Because neurons in the thalamus exhibit unique firing properties, we evaluated the effect of AICP on properties of MDT neurons using current-clamp recordings. No significant changes in spike properties of MDT neurons were observed following application of AICP (supplementary Figure 2; *P*2 > .05; 2-way ANOVA with Bonferroni’s test).

### AICP-Mediated Facilitation of Fear Extinction Involves an Increase in Recruitment of GluA1 AMPA Receptors at BLA Synapses

**Figure 5.**
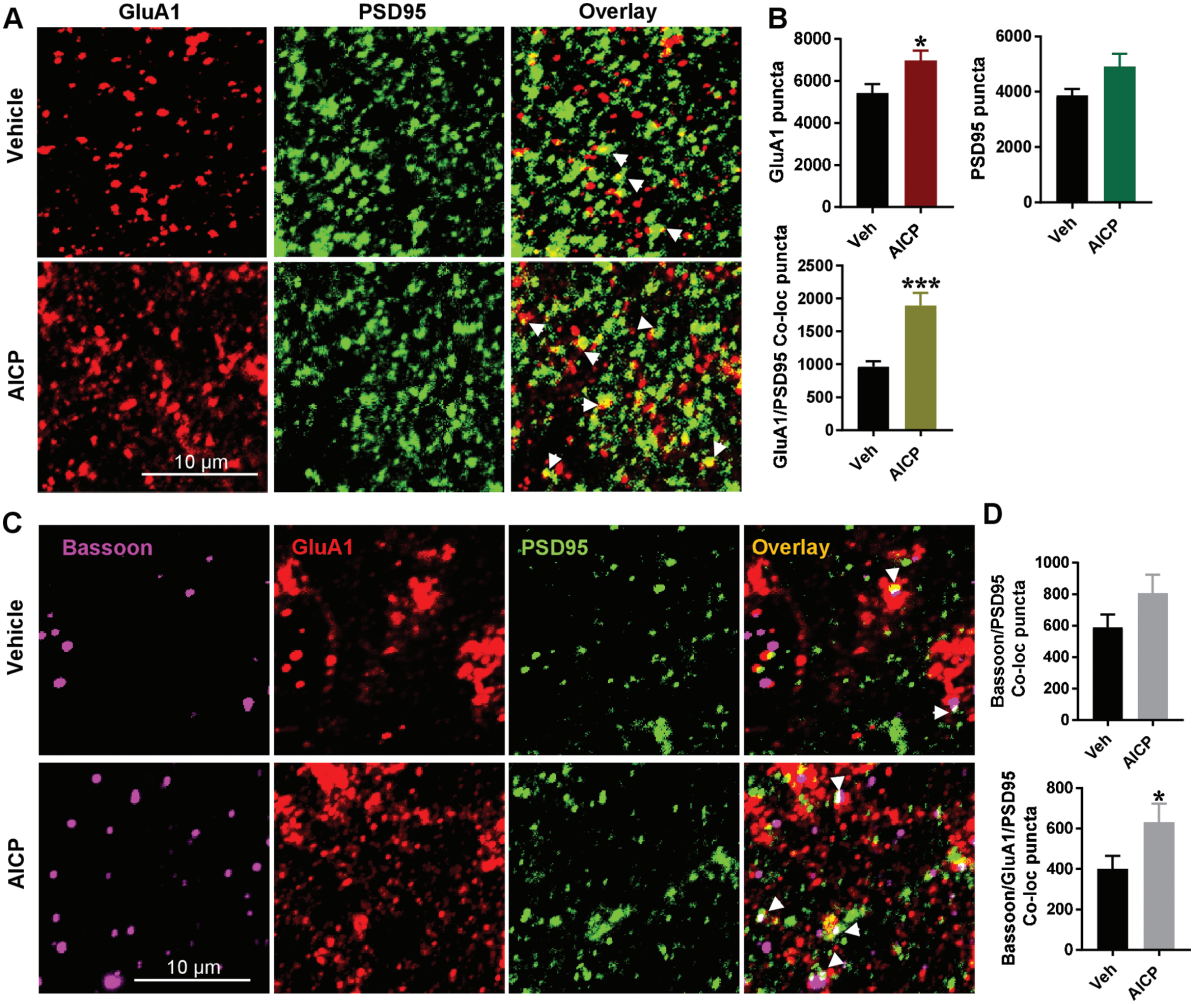
AICP treatment immediately after fear extinction increases synaptic α-amino-3-hydroxy-5-methyl-4-isoxazole propionic acid (AMPA) receptors. (A) Confocal images from basolateral amygdala (BLA) of vehicle-extinction (top) and AICP extinction (100 ng/side, i-BLA, bottom) WT mice immunostained for AMPA receptor GluA1 subunit and PSD 95. Arrowheads showing colocalized puncta. (B) Quantification of immunostaining; AICP treatment led to a significant increase in total GluA1 puncta (*P* = .0196). Trend for an increase in PSD 95 immunoreactive puncta in AICP-extinction group (*P* = .0926). Significant increase in total GluA1-PSD 95 colocalized puncta in AICP-extinction group (*P* = .0002). (C) Confocal images from BLA of vehicle-extinction (top) and AICP-extinction (bottom) WT mice immunostained for bassoon, GluA1 and PSD 95. Arrowheads show colocalized puncta. (D) Quantification of immunostaining. No significant change in bassoon-PSD 95 colocalized puncta by AICP (*P* = .1387). Significant increase in bassoon/GluA1/PSD 95 colocalized puncta (*P* = .0282) by AICP treatment. Bar graphs represent mean ± SEM, each data point representing 22–24 images from n = 3 vehicle and n = 5 AICP mice. Data were analyzed by unpaired *t* test.

In addition to the short-term effect on excitatory neurotransmission, we examined whether AICP produces changes that represent synaptic plasticity. Different mechanisms for DCS action on fear extinction have been identified, which include enhanced inhibitory learning and depotentiation mediated by α-amino-3-hydroxy-5-methyl-4-isoxazole propionic acid (AMPA) receptor endocytosis. We have previously shown an increase in amygdala GluA1 subunit expression by DCS when given in conjunction in fear extinction, suggesting facilitation of synaptic plasticity (Gupta et al., 2013). We evaluated the potential molecular correlate underlying facilitation of fear extinction by AICP using immunohistochemical analysis of synaptic GluA1 expression. Co-labeling for GluA1 and postsynaptic density marker PSD 95 was performed in tissue from vehicle- and AICP-treated animals, and single and co-localized puncta were analyzed in the BLA using confocal imaging (Figure 5A–B). AICP led to a significant increase in total GluA1 puncta (vehicle: 5431 ± 417.8; n = 21 images from 3 mice, AICP: 6977 ± 480.8; n = 23 images from 5 mice, *P* = .0196, unpaired *t* test; Figure 5B) as well as GluA1-PSD 95 colocalized puncta (vehicle 961.6 ± 83.15, AICP 1892 ± 196.1; *P* = .0002, unpaired *t* test; Figure 5B) compared with the vehicle group. We also conducted triple labeling with both pre- and post-synaptic markers bassoon and PSD 95 together with GluA1 to further address changes in number of synapses and GluA1-containing synapses by AICP (Figure 5C–D). A trend for an increase in total number of synapses indicated by bassoon-PSD 95 co-localized puncta was seen in the AICP group (*P* = .1387, unpaired *t* test; Figure 5D). Importantly, GluA1 localized to bassoon-PSD95 elements was also higher in the AICP group (vehicle 400.5 ± 63.5, AICP 607.8 ± 91.49; *P* = .0704, unpaired *t* test; Figure 5D). These results suggest an increase in synaptic efficacy and facilitation of inhibitory learning by AICP. Thus, our immunohistochemistry together with behavioral results suggest that AICP by increasing the function of astrocytic NMDA receptors enhances synaptic plasticity in BLA neurons, which may contribute to facilitation of fear extinction.

## Discussion

There are several key findings from our studies. First, we have identified that a novel glycine-site agonist AICP facilitated fear extinction in a mouse model. This effect of AICP was dependent on the GluN2C subunit in the amygdala since the facilitation of fear extinction was absent in the GluN2C KO. Secondly, we identified that facilitation of fear extinction by AICP was dependent on astrocytic NMDA receptors, since in mice with conditional ablation of the obligatory GluN1 from astrocytes, AICP was ineffective. Complementary chemogenetic experiments further supported the hypothesis that activation of BLA astrocytes can enhance consolidation of fear extinction memory. Acutely, AICP was found to enhance excitatory neurotransmission by a presynaptic mechanism consistent with its potential effect on astrocytic NMDA receptors in the BLA. Finally, our results suggest that increase in synaptic insertion of GluA1 may serve as a molecular correlate for the facilitation of fear extinction by AICP.

### Identification of Novel Glycine Site Agonists With GluN2C-Selective Efficacy

Mechanisms underlying fear extinction have been shown to involve consolidation of memory that require activation of NMDA receptors (Walker et al., 2002; Walker and Davis, 2002; Szinyei et al., 2003; Bauer and LeDoux, 2004). Facilitation of NMDA receptors by DCS has been found to enhance fear extinction (Walker et al., 2002; Walker and Davis, 2002; Gupta et al., 2013). Furthermore, an allosteric potentiator of GluN2C/2D-containing receptors CIQ has been found to facilitate fear extinction (Ogden et al., 2014). Our study dissects the precise subunit-specific (GluN2C or GluN2D) and ensuing cellular mechanism in fear extinction using novel pharmacological and genetic tools. Recently, AICP, a glycine-site agonist with superagonism at GluN2C-containing receptors, has been identified (Jessen et al., 2017) and found to produce GluN2C-dependent in vivo effects in NMDA channel blocker-induced behaviors (Liu et al., 2019). We found that ICV or intra-BLA injections of AICP significantly facilitated fear extinction in WT but not in GluN2C KO mice. We previously found that deletion of GluN2C subunit does not significantly affect fear extinction (Hillman et al., 2011). Thus, based on our previous and current findings, acute facilitation of GluN2C-containing receptors contributes to synaptic plasticity and enhances fear extinction, but constitutive deletion does not impair fear extinction. We also compared the effects of DCS vs AICP on fear extinction in the short extinction training protocol to limit training-induced extinction and evaluate robustness of the pharmacological effect. DCS did not facilitate fear extinction compared with AICP, consistent with higher efficacy of AICP for GluN1/GluN2C receptors compared with other GluN2-containing receptors (Jessen et al., 2017). Future studies with additional doses of DCS and AICP will be needed to directly compare the effectiveness of the 2 drugs on fear extinction. We also found that AICP increased the frequency but not the amplitude of mEPSC in BLA pyramidal neurons, and this effect was absent in GluN2C KO mice. Thus, AICP increases excitatory neurotransmission via a presynaptic mechanism consistent with an effect mediated by astrocytic GluN2C subunit effect. Overall, these results demonstrate that enhancing the function of GluN2C-containing receptors in the BLA may serve as a mechanism for facilitation of fear extinction. However, there are some limitations to the interpretation of these results due to the effect of AICP at other GluN2-containing receptors. AICP inhibits GluN2B- and GluN2D-containing receptors (Jessen et al., 2017). Inhibition of GluN2B in the BLA is known to prevent acquisition of fear extinction (Sotres-Bayon et al., 2007; Dalton et al., 2012); thus, AICP effects are unlikely due to effects on GluN2B-containing receptors. The role of GluN2D-containing receptors in fear extinction is unclear, and their potential contribution to AICP effect will need to be evaluated in future studies.

### Augmentation of Fear Extinction by Facilitation of Amygdala Astrocytes

Previous reports have demonstrated conclusively that astrocytes in cortex and other limbic regions express NMDA receptors (Farb et al., 1995; Conti et al., 1996; Schipke et al., 2001; Karavanova et al., 2007; Cahoy et al., 2008; Hillman et al., 2011; Dzamba et al., 2013; Zhang et al., 2014, 2016; Gokce et al., 2016; Ravikrishnan et al., 2018; Alsaad et al., 2019). Electrophysiology recordings from labeled astrocytes in acute brain slices and singly isolated astrocytes demonstrate that they express NMDA receptor currents (Lalo et al., 2006, 2011; Palygin et al., 2011). These currents are sensitive to GluN2C/2D-selective inhibitors. They also exhibit lower sensitivity to Mg^2+^-block and lack desensitization, which are characteristics of GluN2C/2D-containing receptors (Lalo et al., 2006; Palygin et al., 2011). We have recently demonstrated using a novel reporter model that GluN2C subunit is expressed in several corticolimbic regions, including amygdala in astrocytes (Ravikrishnan et al., 2018). However, the function of NMDA receptors in astrocytes remains unknown. For the first time, to our knowledge, using genetic models and novel pharmacological tools, we demonstrate a role of astrocytic NMDA receptors in the BLA in behavioral plasticity. Using a conditional KO strategy, we deleted the obligatory NMDA receptor GluN1 subunit from astrocytes. This deletion led to loss of the effect of AICP on fear extinction. Control experiments in which the cre cassette was not turned on showed robust facilitation of fear extinction by AICP. Direct activation of BLA astrocytes using a DREADD-Gq signaling following extinction training also facilitated fear extinction, further supporting the regulatory influence of BLA astrocytes. It should be noted that deletion of astrocytic NMDA receptors alone did not affect fear conditioning or fear expression, suggesting that NMDA receptors are not obligatory for these fear-learning processes. Because of the short extinction training paradigm used in our studies, we cannot conclude the precise influence of astrocytic NMDA receptors on within-session extinction, but based on the effect of AICP it can be concluded that astrocytic NMDA receptors influence fear extinction when their function is enhanced. Future studies are necessary to understand the role of astrocytic NMDA receptors in different forms of behavioral plasticity. Indeed, given the widespread distribution of GluN2C in astrocytes in cortico-limbic regions, it is possible that they may contribute significantly to synaptic plasticity and behavioral control.

### Molecular Mechanism of AICP Effect on Fear Extinction

It is widely accepted that extinction learning involves the formation of new memory traces to a novel stimulus (CS+)–outcome (US−) association without obliterating the original CS+ – US+ association (Bouton, 2004; Hartley and Phelps, 2010). NMDA receptor–dependent plasticity in BLA has been shown to play an important role in the acquisition of fear extinction (Walker et al., 2002; Walker and Davis, 2002). Moreover, increasing NMDA receptor function by DCS also facilitates fear extinction (Walker et al., 2002; Mao et al., 2006). Similarly, in the present study, AICP was found to facilitate extinction. The molecular mechanism for DCS effect on fear extinction has been shown to involve AMPA receptor endocytosis, and a reversal of conditioning induced increased GluA1 surface expression (Mao et al., 2006). However, we previously found evidence that supports a role of DCS in formation of new inhibitory learning memory. DCS in conjunction with fear extinction training increases GluA1 expression in the amygdala (Gupta et al., 2013). These differences in molecular effects of increasing NMDA receptor activity may potentially depend on the specific extinction paradigm. A stronger extinction training may initiate erasure (Mao et al., 2006), whereas a milder extinction training as in the present study may induce new inhibitory learning. Consistent with our previous finding (Gupta et al., 2013), we show here that AICP increases the synaptic insertion of GluA1 subunit. However, it remains unknown how astrocytic NMDA receptors may contribute to synaptic plasticity at neuronal synapses. Some potential mechanisms may involve increase in intracellular Ca^2+^ (Palygin et al., 2010) and downstream release of gliotransmitters (Araque et al., 2014; Alsaad et al., 2019) or synaptogenic molecules (Allen and Eroglu, 2017) that may increase synaptic plasticity. It has been previously shown that activation of astrocytic NMDA receptors leads to an increase in intracellular calcium in astrocytes. Additionally, stimulation of neuronal afferents in cortical slices increases cytosolic Ca^2+^ in astrocytes, which were partially blocked by NMDA receptor antagonists (Palygin et al., 2010). It has been found that activation of astrocytic signaling can cause release of glutamate or D-serine, which may increase excitatory neurotransmission (Araque et al., 2014; Alsaad et al., 2019). Indeed, our results using AICP and GluN2C KO model suggest that in brain slices, activation of NMDA receptors on astrocyte increases excitatory neurotransmission via a presynaptic mechanism, which may involve gliotransmitter release. Thus, activation of astrocytic NMDA receptors may serve as a feedback mechanism to further potentiate synapses engaged during extinction learning by release of gliotransmitters. With regard to astrocytic synaptogenic factors, it has been found that astrocytes release glypican 4 and 6, which increase surface expression of GluA1 (Allen et al., 2012; Farhy-Tselnicker et al., 2017). Therefore, the possibility exists that AICP, via facilitation of astrocytic GluN2C-containing NMDA receptors, may induce the release of astrocyte-secreted factors, which results in an increase in synaptic GluA1. Finally, we found that the effect of astrocytic NMDA receptors was specific to modulation of fear extinction. AICP facilitated fear extinction but did not affect fear conditioning. This suggests that the synaptic plasticity events that underlie fear conditioning and extinction may engage amygdala astrocytic NMDA receptors differently, leading to specific behavioral outcome. Another scenario may involve heterogeneity of expression of GluN2C-containing NMDA receptors in astrocytes such that it affords specificity to extinction vs fear-learning engrams. Such astrocytic heterogeneity has been identified in the dorsal striatum wherein astrocytes play distinct roles in direct and indirect pathway medium spiny neurons as well as at cortical vs thalamic synapses (Dvorzhak et al., 2018). Overall, our work demonstrates for the first time, to our knowledge, the contribution of astrocytic NMDA receptors in behavioral control. Future studies are necessary to better understand the synaptic and cellular mechanisms underlying behavioral control by astrocytic NMDA receptors.

## Supplementary Material

pyab055_suppl_Supplementary_Information
