# Supplementary material for: Astrocytic NMDA Receptors in the Basolateral Amygdala Contribute to Facilitation of Fear Extinction
Source: Int J Neuropsychopharmacol. 2021 Aug 7;24(11):907–19. doi: 10.1093/ijnp/pyab055 (PMC8598288; doi:10.1093/ijnp/pyab055)
Supplement: pyab055_suppl_Supplementary_Information [file pyab055_suppl_supplementary_information.docx]

**Astrocytic NMDA receptors in the basolateral amygdala contribute to facilitation of fear extinction**

**Extended data**

**Supplementary figure 1:**


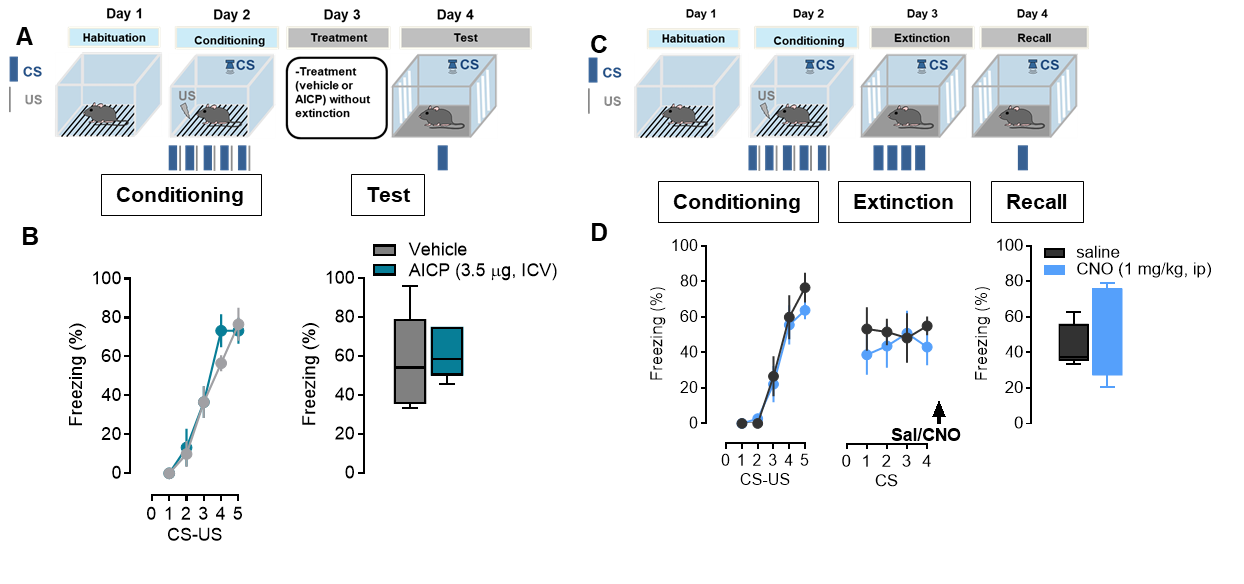


**Supplementary Figure 1. Effect of AICP and CNO on fear recall test. A.** Experimental design used for fear conditioning and recall test. CS, conditioned stimulus; US, unconditioned stimulus. **B.** ICV injection of AICP (3.5 µg) without extinction training did not show significant effect on freezing behavior in recall test in WT animals (vehicle 56.66 ± 11.15 vs AICP 61.66 ± 5.804; *p* = 0.7045; N = 5 vehicle, 5 AICP; unpaired-t test). **C**. Experimental design used for fear conditioning extinction and recall test. CS, conditioned stimulus; US, unconditioned stimulus. **D**. Treatment of CNO immediately after extinction training in no DREADD injected mice did not show any significant effect on freezing behavior in post-extinction recall test (saline 44.17 ± 5.369 vs CNO 52.08 ± 9.668; *p* = 0.4955; N = 5 vehicle, 6 AICP; unpaired-t test).

**Supplementary figure 2:**


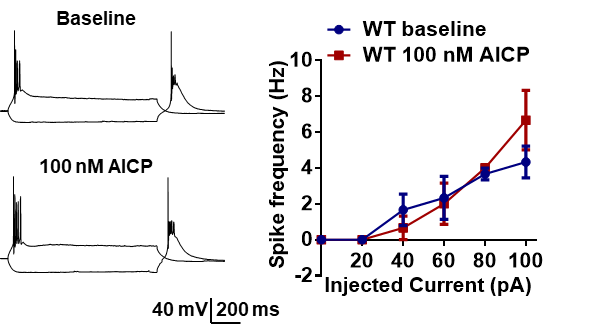


**Supplementary Figure 2. Application of AICP does not affect spike frequency of nRT neurons.** Current-clamp recordings were obtained from MDT neurons and effect of depolarizing current injection on spike frequency was evaluated. No change in the spike frequency was observed following application of AICP (*p* > 0.05, two-way ANOVA with Bonferroni’s post hoc test).
